# Enabling Conducting Polymer Applications: Methods for Achieving High Molecular Weight in Chemical Oxidative Polymerization in Alkyl- and Ether-Substituted Thiophenes

**DOI:** 10.3390/ma14206146

**Published:** 2021-10-16

**Authors:** David D. Hebert, Michael A. Naley, Carter C. Cunningham, David J. Sharp, Emma E. Murphy, Venus Stanton, Jennifer A. Irvin

**Affiliations:** 1 Department of Chemistry and Biochemistry, Texas State University, San Marcos, TX 78666, USA; hebertdavidd@gmail.com (D.D.H.); ccc179@txstate.edu (C.C.C.); davidsharp97@gmail.com (D.J.S.); emmamurphy5693@gmail.com (E.E.M.); v_s136@txstate.edu (V.S.); 2 Department of Biology, Texas State University, San Marcos, TX 78666, USA; mnaley6@gmail.com; 3 Materials Science, Engineering and Commercialization Program, Texas State University, San Marcos, TX 78666, USA

**Keywords:** poly(3-hexylthiophene), polythiophenes, oxidative polymerization, gel-permeation chromatography, high molecular weight, conductive polymers, order of addition, iron (III) chloride, alkyl-substituted EDOT, 3,4-dialkoxythiophene

## Abstract

Polythiophenes (PTs) constitute a diverse array of promising materials for conducting polymer applications. However, many of the synthetic methods to produce PTs have been optimized only for the prototypical alkyl-substituted example poly(3-hexylthiophene) (P3HT). Improvement of these methods beyond P3HT is key to enabling the widespread application of PTs. In this work, P3HT and two ether-substituted PTs poly(2-dodecyl-2H,3H-thieno[3,4-b][1,4]dioxine) (PEDOT-C12) and poly(3,4-bis(hexyloxy)thiophene) (PBHOT) are synthesized by the FeCl_3_-initiated oxidative method under different conditions. Polymerization was carried out according to a common literature procedure (“reverse addition”) and a modified method (“standard addition”), which differ by the solvent system and the order of addition of reagents to the reaction mixture. Gel-permeation chromatography (GPC) was performed to determine the impact of the different methods on the molecular weights (M_w_) and degree of polymerization (X_w_) of the polymers relative to polystyrene standards. The standard addition method produced ether-substituted PTs with higher M_w_ and X_w_ than those produced using the reverse addition method for sterically unhindered monomers. For P3HT, the highest M_w_ and X_w_ were obtained using the reverse addition method. The results show the oxidation potential of the monomer and solution has the greatest impact on the yield and X_w_ obtained and should be carefully considered when optimizing the reaction conditions for different monomers.

## 1. Introduction

Polythiophenes are among the most widely researched classes of conducting polymers, owing to their remarkable stability towards oxygen and moisture [1,2]. Their unique optoelectronic properties have made them of much interest for applications, including polymer solar cells [3,4], transistors [5], chemical sensors [6], and light-emitting diodes [7]. Soluble polythiophenes possess the additional benefit of being processable via solution and printing techniques [8,9], which is advantageous for large-scale manufacturing. In order to enable the use of polythiophenes in a wide range of applications, synthetic approaches that can produce bulk quantities of soluble conducting polymers are necessary.

**Scheme 1:**
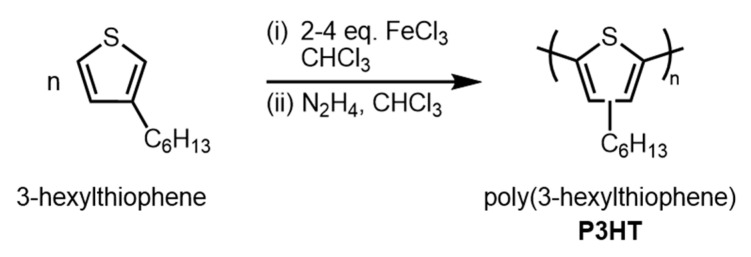
FeCl_3_-initiated oxidative polymerization of 3-hexylthiophene.

To date, numerous synthetic approaches to polythiophenes have been described, including electrochemical [10], chemical oxidative [11], and transition metal-mediated polymerization [4,12,13,14]. Among this abundance of possibilities, the FeCl_3_-initiated oxidative polymerization followed by reduction to the neutral (undoped) polymer (Scheme 1) remains a valuable tool for the synthesis of polythiophenes since it was initially described by Sugimoto et al. in the mid-1980s [11]. Compared to electrochemical and organometallic approaches, the method is convenient, low cost, and can be performed on large scales [15].

Poly(3-hexylthiophene) (P3HT) was one of the first polymers reported to be synthesized using the modern FeCl_3_-initiated polymerization method, and it has become one of the most extensively researched polythiophenes. Polymerization methodologies for P3HT are well-established, and in many contexts, it may be classified as a model system. For example, with the FeCl_3_-initiated polymerization method, high molecular weight (>70,000 g/mol) P3HT with regioregularity of 70–90% is readily obtainable in good yields [16,17,18,19,20,21]. However, outside of 3-hexylthiophene and other closely related alkylthiophenes, polymerization conditions often require significant optimization to achieve similar yields and molecular weights. Given the wide scope of conjugated polymer applications, P3HT alone is insufficient in meeting the needs of every application.

**Figure 1:**
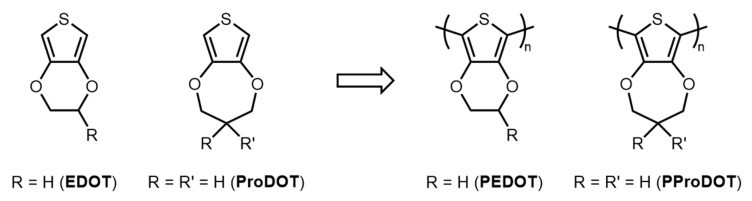
General monomer (left of arrow) and polymer (right of arrow) structures of 3,4-ethylenedioxythiophene (EDOT) and 3,4-propylenedioxythiophene (ProDOT) and their functionalized derivatives.

3,4-Alkylenedioxythiophene monomers, such as 3,4-ethylenedioxythiophene (EDOT) and 3,4-propylenedioxythiophene (ProDOT), have proven to be highly versatile platforms for functional conductive polymers (Figure 1) [22,23,24,25,26,27,28] and present many advantages over 3-hexylthiophene. Namely, the electron-donating ether substituents lower the polymer’s oxidation potential and increase its stability in the doped state [29,30]. Additionally, unlike 3-hexylthiophene, EDOT and ProDOT have the advantage of substitution at both the 3 and 4 positions, thereby eliminating the possibility of β-coupling during polymerization, which leads to poorly defined, insoluble materials.

A survey of the literature reveals that ether-substituted polythiophenes synthesized by FeCl_3_-initiated oxidative polymerization rarely achieve the degree of polymerization or molecular weight reported for P3HT (Table 1) [31,32]. Methods to improve these polymer’s molecular weights and regioregularities are of interest because polymers with higher molecular weight and regioregularity exhibit improved thermal properties [33], optical properties [34], and carrier mobilities [35].

It is important to note that there is considerable variability in the molecular weights reported for polymers prepared under similar conditions [20,36], for example, P3HT synthesized in chloroform with four equivalents of FeCl_3_ (entries 2 and 3 in Table 1). Some of this variability can be attributed to discrepancies between the amounts of solvent used and whether the solvent contains a radical inhibitor (for example, ethanol in chloroform).

**Table 1:** Previously reported molecular weight data of P3HT and relevant alkyl and ether-substituted polythiophenes synthesized by FeCl_3_-initiated oxidative polymerization ^1^.

Entry	Structure	Acronym	Equivalents FeCl_3_	M_w_^2^	X_w_^3^	Ref.
1	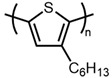	P3HT	2	140,000	842	[16]
2			4	110,700	666	[18]
3			4	411,000	2472	[19]
4	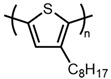	P3OT	4	181,440	933	[37]
5	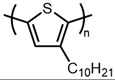	P3DT	4	303,050	1362	[37]
6	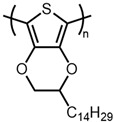	PEDOT-C14	2	11,200	33	[26]
7			4	22,500	67	[26]
8	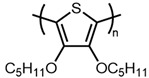	PBPOT	4	9743	38	[38]
9	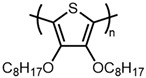	PBOOT	4	11,528	38	[38]

^1^ All polymerizations were performed under reverse addition conditions (monomer added to oxidant). ^2^ Weight-average molecular weight (M_w_) of the neutral (undoped) polymers in g/mol. ^3^ Weight-average degree of polymerization (X_w_ = M_w_/M_0_, where M_0_ is the molecular weight of the repeat unit).

Several studies have been conducted to examine the effects of varying different reaction parameters. Varying the reaction temperature, solvent, monomer concentration, and monomer/oxidant ratio can affect reaction yields as well as polymer regioregularity and molecular weight (summarized in Table 2) [16,17,18,19,39,40,41,42]. Lower reaction temperatures improve polydispersity at the cost of a slight reduction in yield [40]. Polymers prepared in better solvents tend to have higher molecular weight and improved regioregularity [40]. Reducing the ratio of oxidant to monomer sharply decreases yields, and at sub-stoichiometric amounts, molecular weight is severely impacted [16].

**Figure 2:**
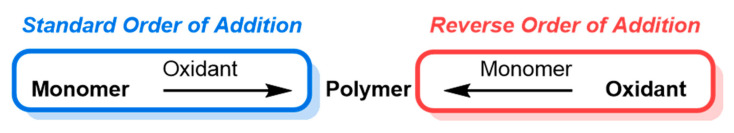
Differences between standard and reverse addition in chemical oxidative polymerization.

In this work, we examine the FeCl_3_-initiated oxidative polymerization method and the impact of the order of reagent addition on the molecular weight and degree of polymerization of 3-hexylthiophene, an alkyl-substituted PEDOT, and an alkoxy-substituted polythiophene. Among the many studies on the oxidative polymerization reaction, the order of addition of reagents is rarely considered. The reaction can be performed using what we term “standard” or “reverse” order of addition (Figure 2). Under standard conditions, the oxidant is slowly added to the monomer. Under reverse conditions, the opposite occurs. The original publication by Sugimoto et al. [11] describes polymerization of 3-hexylthiophene under reverse addition conditions, and the bulk of other studies are carried out in this fashion [16,17,18,19,26,31,36,37,38,40,43,44,45].

**Figure 3:**
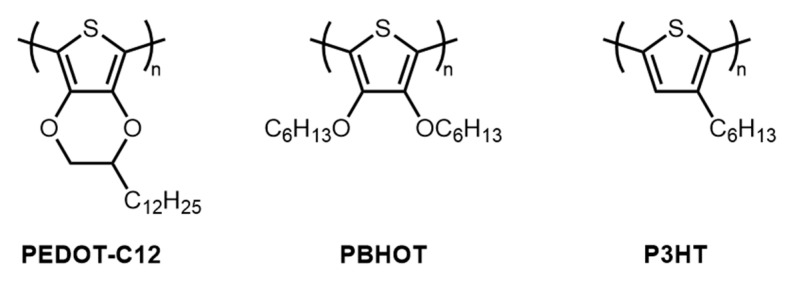
The polymers examined in this work.

There are relatively few papers describing the reaction under standard addition conditions [15,21,46]. This could be due in part to the poor solubility of FeCl_3_ in solvents, such as chloroform and chlorobenzene, which are good solvents for polythiophenes. Thus, it is typically more convenient to add a monomer solution to a flask containing a suspension of FeCl_3_ than vice versa. Preparing an oxidant suspension that is easily handled and suitable for use over an extended period (e.g., slow addition over several minutes) typically requires the use of sonication [16,17]. An alternative is to simply perform the reaction in a good solvent for the oxidant. However, the polar solvents in which FeCl_3_ is soluble are typically poor solvents for polythiophenes, which can lead to significantly decreased molecular weights [19,36]. Herein, we describe a modified standard addition method that addresses these issues and evaluate the impact of order of addition, solvent composition, oxidant concentration, and reaction time on degree of polymerization of two different ether-substituted polythiophenes, poly(2-dodecyl-2H,3H-thieno [3,4-b][1,4]dioxine) (PEDOT-C12) and poly(3,4-bis(hexyloxy)thiophene) (PBHOT) in comparison with P3HT (Figure 3).

## 2. Materials and Methods

### 2.1. General

Glassware was dried in an oven prior to use unless noted otherwise. Molecular sieves (4 Å) were activated by first drying at 200 °C under vacuum in a vacuum-oven for 24 h, then quickly transferred to a Schlenk flask (Chemglass Life Sciences, Vineland, NJ, USA) and flame-dried under high vacuum several times. The sieves were kept under high vacuum for 6 h before use. Chloroform (ACS grade, Avantor, Radnor Township, PA, USA) and chlorobenzene (99%, Alfa Aesar, Haverhill, MA, USA) were dried over activated 4 Å molecular sieves and protected from light and used rapidly. The following chemicals were used as received: anhydrous FeCl_3_ (98%, Alfa Aesar), anhydrous hydrazine (98%, Sigma Aldrich, St. Louis, MO, USA), methanol (HPLC grade, J.T. Baker, Radnor Township, PA, USA), acetonitrile (99.9%+, Acros Organics, Geel, Belgium), 3-hexylthiophene (>98%, TCI Chemicals, Tokyo, Japan), 1,2-tetradecanediol (90%, Sigma Aldrich), anhydrous *n*-hexanol (≥99%, Sigma Aldrich), 3,4-dimethoxythiophene (97%, Ark Pharm Inc., Arlington Heights, IL, USA), and anhydrous toluene (99.8%, Sigma Aldrich). Monomer characterization details can be found in the Supplementary Materials, including ^1^H NMR spectra of EDOT-C12 (Figure S1) and BHOT (Figure S2). GPC experimental information, including a calibration curve (Figure S4) and elugrams for all polymers (Figures S5–S7), can also be found in the Supplementary Materials.

### 2.2. Monomer Synthesis

**Scheme 2:**
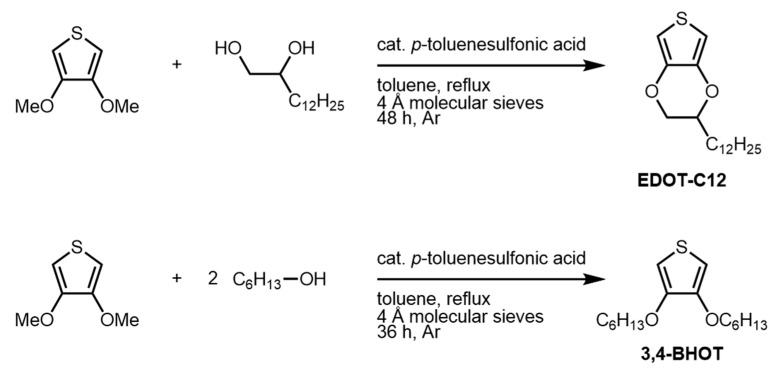
Synthesis of ether-substituted thiophene monomers.

Alkyl-substituted EDOT monomer 2-dodecyl-2H,3H-thieno[3,4-b][1,4]dioxine (EDOT-C12) and alkoxy-substituted monomer 3,4-bis(hexyloxy)thiophene (3,4-BHOT) were synthesized using a modified literature procedure via *p*-toluenesulfonic acid-catalyzed transetherification [47,48] of 3,4-dimethoxythiophene and the corresponding alcohol (Scheme 2). Monomer EDOT-C12 was synthesized using one equivalent of 1,2-tetradecanediol, and monomer 3,4-BHOT was synthesized using two equivalents of *n*-hexanol.

#### 2.2.1. Synthesis of EDOT-C12

To a 1 L three-necked round bottom flask (Chemglass Life Sciences) outfitted with a magnetic stir bar (Fisher Scientific, Hampton, NH, USA), Soxhlet extractor (Chemglass Life Sciences) charged with 4 Å molecular sieves and high-efficiency condenser Chemglass Life Sciences) was added 1,2-tetradecanediol (17.59 g, 76.3 mmol) and *p*-toluenesulfonic acid (1.33 g, 7.0 mmol) against a positive pressure of argon. Toluene (400 mL) was added, and the flask was sealed with a rubber septum. Stirring was initiated, and the flask was heated at 60 °C. Once all solids had dissolved, the septum was removed and 3,4-dimethoxythiophene (DMT, 10.00 g, 87.6 mmol) was added against a positive pressure of argon. The flask was resealed, and the mixture was heated at 120 °C for 48 h under argon. The colorless mixture slowly darkened to dark brown over several hours after addition of the DMT. The mixture was then cooled to room temperature and poured into a 1 L separatory funnel (Fisher Scientific). The crude reaction mixture was washed 4 times with portions (ca. 200 mL each) of deionized water. The organic fraction was collected, dried over anhydrous MgSO_4_, and filtered. The filtrate was evaporated under reduced pressure to give the crude product a dark brown oil. The crude product was purified by filtration through silica gel with hexanes followed by removal of the solvent under reduced pressure. The yellow solid was recrystallized from diethyl ether at −78 °C to give 6.02 g (25%) product as a slightly yellow powder. ^1^H NMR (Figure S1, 400 MHz, CDCl_3_) δ: 6.30 (s, 2H), 4.14 (dd, *J* = 11.3, 2.1 Hz, 1H), 4.10 (m, 1H), 3.86 (dd, *J* = 11.3, 7.9 Hz, 1H), 1.67–1.27 (m, 22H), 0.89 (t, 3H); Lit. [23]: 6.30 (s, 2H), 4.12 (m, 2H), 3.86 (m, 1H), 1.40 (m, 22H), 0.88 (t, 3H); MS (Figure S3, *m*/*z*): [M + H]^+^ calcd for C_18_H_31_O_2_S^+^ 311.204; found 311.167.

#### 2.2.2. Synthesis of 3,4-BHOT

A three-necked round bottom flask was outfitted with Soxhlet extractor charged with activated 4 Å molecular sieves, high efficiency condenser, and magnetic stir bar. Toluene (100 mL) and *p*-toluenesulfonic acid (0.40 g, 2.1 mmol) were added to the flask, and stirring was initiated. The flask was sealed with a rubber septum and heated at 60 °C under argon. The mixture was stirred for 5–10 min at that temperature, and *n*-hexanol (5.78 mL, 46.0 mmol) was added through the septum via syringe. After an additional 5 min, 3,4-dimethoxythiophene (3.00 g, 26.3 mmol) in toluene (5 mL) was added slowly through the septum via syringe. The mixture was heated for 36 h at 130 °C under argon. The mixture was cooled to room temperature and transferred to a 500 mL separatory funnel. The crude reaction mixture was washed three times with deionized water (60 mL each), once with sat. NaHCO_3_ (60 mL), and once again with water (60 mL). The organic fraction was collected, dried over anhydrous MgSO_4_, and filtered. The filtrate was evaporated under reduced pressure to give the crude product as a brown oil. The crude product was purified by short-path vacuum distillation. Yield 4.51 g (69%) product as a light-yellow oil. ^1^H NMR (Figure S2, 400 MHz, CDCl_3_) δ: 6.16 (s, 2H), 3.98 (t, 4H), 1.81 (p, 4H), 1.44 (m, 4H), 1.33 (m, 8H), 0.90 (t, 6H); Lit. [49]: 6.15 (s, 2H), 3.98 (t, 4H), 1.81 (m, 4H), 1.35–1.43 (m, 12H), 0.97 (t, 6H).

### 2.3. Polymerizations

#### 2.3.1. General Procedure—Reverse Addition Oxidative Polymerization

A typical reverse addition polymerization procedure was conducted as follows: anhydrous FeCl_3_ (2.3 or 4 molar equivalents relative to monomer) was weighed and quickly transferred to a Schlenk flask. The flask was purged with argon, and dry chlorobenzene (25 mL) was added. The oxidant suspension was stirred rapidly for 3–5 min under argon. Monomer (0.7–1.6 mmol) was dissolved in dry chlorobenzene (5 mL) and added dropwise to the stirred oxidant suspension via syringe (BD, Franklin Lakes, NJ, USA). The mixture was stirred for 24 h at room temperature, after which the polymer was precipitated by dropwise addition of the reaction mixture into an excess (250 mL) of rapidly stirred methanol. The polymer was collected by vacuum filtration and washed thoroughly with methanol. The polymer was allowed to air dry before being resuspended in chlorobenzene (25–40 mL) under argon and reduced by the addition of anhydrous hydrazine (1–2 molar equivalents relative to monomer) via syringe. The mixture was stirred for 24 h at room temperature, after which the polymer was precipitated into excess methanol, collected by filtration, and washed as above. The polymer was dried under vacuum and stored under argon in the dark.

#### 2.3.2. PEDOT-C12—Reverse Addition, 2.3 Equivalents FeCl_3_ (Table 3, Entry 1)

A solution of EDOT-C12 (251 mg, 0.8 mmol) in chlorobenzene (6 mL) was added to a suspension of anhydrous FeCl_3_ (314 mg, 1.9 mmol) in chlorobenzene (24 mL), resulting in a dark blue mixture. The mixture was stirred for 24 h, and the polymer was precipitated and collected by filtration. The polymer was resuspended in chlorobenzene (25 mL), and anhydrous hydrazine (0.03 mL, 1.0 mmol) was added, causing a gradual color change of the solution to violet. The polymer was purified as described above. Yield 183 mg (73%) product as a dark violet powder.

#### 2.3.3. PEDOT-C12—Reverse Addition, 2.3 Equivalents FeCl_3_ in Chloroform (Table 3, Entry 2)

A solution of EDOT-C12 (252 mg, 0.8 mmol) in chloroform (6 mL) was added to a suspension of anhydrous FeCl_3_ (326 mg, 2 mmol) in chloroform (24 mL), resulting in a dark blue mixture. The mixture was stirred for 24 h, and the polymer was precipitated and collected by filtration. The polymer was resuspended in chloroform (25 mL), and anhydrous hydrazine (0.03 mL, 1.0 mmol) was added, causing a gradual color change of the solution to violet. The polymer was purified as described above. Yield 200 mg (80%) product as a dark violet powder.

#### 2.3.4. PBHOT—Reverse Addition, 2.3 Equivalents FeCl_3_ (Table 3, Entry 5)

A solution of 3,4-BHOT (219 mg, 0.8 mmol) in chlorobenzene (6 mL) was added to a suspension of anhydrous FeCl_3_ (295 mg, 1.8 mmol) in chlorobenzene (30 mL), resulting in a dark green/blue mixture. The mixture was stirred for 24 h, and the polymer was precipitated and collected by filtration. The polymer was resuspended in chlorobenzene (30 mL), and anhydrous hydrazine (0.03 mL, 0.96 mmol) was added, causing a gradual color change of the solution to red. The polymer was purified as described above. Yield 92 mg (42%) product as a red powder.

#### 2.3.5. P3HT—Reverse Addition, 2.3 Equivalents FeCl_3_ (Table 3, Entry 9)

A solution of 3-hexylthiophene (234 mg, 1.4 mmol) in chlorobenzene (6 mL) was added to a suspension of anhydrous FeCl_3_ (525 mg, 3.2 mmol) in chlorobenzene (30 mL), resulting in a dark green mixture. The mixture was stirred for 24 h, and the polymer was precipitated and collected by filtration. The polymer was resuspended in chlorobenzene (30 mL), and anhydrous hydrazine (0.05 mL, 1.6 mmol) was added, causing a gradual color change of the solution to orange. The polymer was purified as described above. Yield 173 mg (75%) product as a dark red powder.

#### 2.3.6. General Procedure—Standard Addition Oxidative Polymerization

A typical standard addition procedure was conducted as follows: anhydrous FeCl_3_ (either 2.3 or 4 molar equivalents relative to monomer) was quickly weighed into a dry 20 mL scintillation vial and sealed with a septum cap. The vial was purged with argon, and acetonitrile (5 mL) was added via syringe to give a dark red solution. The FeCl_3_ solution was added dropwise to a well-stirred solution of monomer (0.7–1.0 mmol) dissolved in 30 mL dry chlorobenzene under argon and stirred for 24 or 48 h at room temperature. The polymer was then precipitated by dropwise addition of the reaction mixture into an excess (250 mL) of rapidly stirred methanol. In certain cases, the reaction mixture had to be concentrated under reduced pressure prior to precipitation due to solubility issues. The polymer was collected by vacuum filtration and washed thoroughly with methanol. The polymer was allowed to air dry before being resuspended in chlorobenzene (25–40 mL) under argon and reduced by the addition of anhydrous hydrazine (1–2 molar equivalents relative to monomer) via syringe. The mixture was stirred for 24 h at room temperature, after which the polymer was precipitated into excess methanol, collected by filtration, and washed as above. The polymer was dried under vacuum and stored under argon in the dark. 

#### 2.3.7. PEDOT-C12—Standard Addition, 2.3 Equivalents FeCl_3_ (Table 3, Entry 3)

A solution of anhydrous FeCl_3_ (312 mg, 1.9 mmol) in acetonitrile (5 mL) was added to a solution of EDOT-C12 (253 mg, 0.8 mmol) in chlorobenzene (30 mL), resulting in a dark green mixture. The mixture was stirred for 24 h, and the polymer was precipitated and collected by filtration. The polymer was resuspended in chlorobenzene (25 mL), and anhydrous hydrazine (0.04 mL, 1.3 mmol) was added, causing a color change of the solution to violet. The polymer was purified as described above. Yield 65 mg (26%) product as a dark purple powder.

#### 2.3.8. PEDOT-C12—Standard Addition, 4 Equivalents FeCl_3_ (Table 3, Entry 4)

A solution of anhydrous FeCl_3_ (435 mg, 2.7 mmol) in acetonitrile (5 mL) was added to a solution of EDOT-C12 (202 mg, 0.7 mmol) in chlorobenzene (30 mL), resulting in a dark green mixture. The mixture was stirred for 24 h, and the polymer was precipitated and collected by filtration. The polymer was resuspended in chlorobenzene (40 mL), and anhydrous hydrazine (0.04 mL, 1.3 mmol) was added, causing a color change from dark blue to violet. The polymer was purified as described above. Yield 116 mg (58%) product as a dark purple powder.

#### 2.3.9. PBHOT—Standard Addition, 2.3 Equivalents FeCl_3_ (Table 3, Entry 6)

A solution of anhydrous FeCl_3_ (330 mg, 2.0 mmol) in acetonitrile (5 mL) was added to a solution of 3,4-BHOT (250 mg, 0.9 mmol) in chlorobenzene (30 mL), resulting in a dark green mixture. The mixture was stirred for 24 h, then concentrated under reduced pressure. The polymer was precipitated and collected by filtration, then resuspended in 35 mL chlorobenzene. Anhydrous hydrazine (0.03 mL, 1.0 mmol) was added, causing a color change to red, and the mixture was stirred for 24 h. Due to solubility issues, this sample was unable to be collected by precipitation and filtration. Instead, the solvent was removed under reduced pressure to provide the product. Yield 195 mg (78%) product as a dark tacky solid.

#### 2.3.10. PBHOT—Standard Addition, 4 Equivalents FeCl_3_ (Table 3, Entry 7)

A solution of anhydrous FeCl_3_ (467 mg, 2.9 mmol) in acetonitrile (5 mL) was added to a solution of 3,4-BHOT (205 mg, 0.7 mmol) in chlorobenzene (30 mL), resulting in a dark green mixture. The mixture was stirred for 24 h, and the polymer was precipitated and collected by slow filtration. The polymer was resuspended in 40 mL chlorobenzene, and anhydrous hydrazine (0.04 mL, 1.3 mmol) was added (note: methanol alone appeared to be sufficient to reduce this polymer as evidenced by the change to a red color during the precipitation step). The mixture was stirred for 24 h, then concentrated under reduced pressure before being purified as described above. Yield 22 mg (11%) product as a blood red powder.

#### 2.3.11. PBHOT—Standard Addition, 4 Equivalents FeCl_3_ 48 h (Table 3, Entry 8)

A solution of anhydrous FeCl_3_ (573 mg, 3.5 mmol) in acetonitrile (5 mL) was added to a solution of 3,4-BHOT (251 mg, 0.9 mmol) in chlorobenzene (30 mL), resulting in a dark green mixture. The mixture was stirred for 48 h, and the polymer was precipitated and collected by filtration. The polymer was resuspended in chlorobenzene (30 mL), and anhydrous hydrazine (0.03 mL, 1.0 mmol) was added, causing the solution to turn dark purple. The mixture was stirred for 24 h, then concentrated under reduced pressure before being purified as described above. Yield 15 mg (10%) product as a dark purple powder.

#### 2.3.12. P3HT—Standard Addition, 2.3 Equivalents FeCl_3_ (Table 3, Entry 10)

A solution of anhydrous FeCl_3_ (521 mg, 3.2 mmol) in acetonitrile (5 mL) was added to a solution of 3-hexylthiophene (234 mg, 1.4 mmol) in chlorobenzene (30 mL), resulting in a dark green mixture. The mixture was stirred for 24 h, and the polymer was precipitated and collected by filtration. The polymer was resuspended in chlorobenzene (40 mL), and anhydrous hydrazine (0.04 mL, 1.3 mmol) was added, causing a color change to bright orange. The polymer was purified as described above. Yield 60 mg (26%) product as a dark red powder.

#### 2.3.13. P3HT—Standard Addition, 4 Equivalents FeCl_3_ (Table 3, Entry 11)

A solution of anhydrous FeCl_3_ (1.03 g, 6.4 mmol) in acetonitrile (5 mL) was added to a solution of 3-hexylthiophene (271 mg, 1.6 mmol) in chlorobenzene (30 mL), resulting in a dark green mixture. The mixture was stirred for 24 h, and the polymer was precipitated and collected by filtration. The polymer was resuspended in chlorobenzene (40 mL), and anhydrous hydrazine (0.04 mL, 1.3 mmol) was added, causing a color change from dark blue to bright orange. The polymer was purified as described above. Yield 67 mg (25%) product as a dark red powder.

#### 2.3.14. P3HT—Standard Addition, 4 Equivalents FeCl_3_ 48 h (Table 3, Entry 12)

A solution of anhydrous FeCl_3_ (973 mg, 6.0 mmol) in acetonitrile (5 mL) was added to a solution of 3-hexylthiophene (252 mg, 1.5 mmol) in chlorobenzene (30 mL), resulting in a dark green mixture. The mixture was stirred for 48 h, and the polymer was precipitated and collected by filtration. The polymer was resuspended in chlorobenzene (30 mL), and anhydrous hydrazine (0.05 mL, 1.6 mmol) was added, causing a color change of the solution to orange. The mixture was stirred for 24 h, concentrated under reduced pressure and purified as above, yielding 179 mg (72%) product as a dark red powder.

## 3. Results and Discussion

The degree of polymerization (X_w_), weight-average molecular weight (M_w_), and yields for the polymers are given in Table 3. We will first consider the polymers prepared using the common literature method (reverse order of addition). The M_w_ and X_w_ of these polymers agree with the trends observed in previously reported data (see Table 1). As expected based on prior reports [50], comparison of Table 3 entries 1 and 2 shows that polymerization of EDOT-C12 is much more effective in chlorobenzene than in chloroform, producing PEDOT-C12 with 20 repeat units in chlorobenzene rather than low molecular weight oligomers in chloroform. Thus, all other polymerizations were conducted in chlorobenzene. The X_w_ of polymers prepared using the reverse order of addition with 2.3 molar equivalents FeCl_3_ was high for P3HT (X_w_ 904, M_w_ 150,000 g/mol), and low for ether-substituted PEDOT-C12 (X_w_ 20, M_w_ 6300 g/mol) and PBHOT (X_w_ < 18, M_w_ <5200 g/mol, outside the calibrated region of the analytical column). Changing the order of addition to standard addition had the most significant impact on X_w_ for PEDOT-C12. Use of the standard addition method with 2.3 equivalents FeCl_3_ resulted in PEDOT-C12 with X_w_ 65 (M_w_ 20,000 g/mol), a three-fold increase over the analogous reverse addition case. However, the same conditions resulted in no observable change in X_w_ for PBHOT (X_w_ < 18, M_w_ <5200 g/mol) and a decrease for P3HT (X_w_ 488, M_w_ 81,000 g/mol) compared to the equivalent reverse addition cases.

When four molar equivalents FeCl_3_ were used with the standard addition method, a sizeable increase in X_w_ was observed for PEDOT-C12 and PBHOT. The X_w_ of PEDOT-C12 prepared with these conditions was 747 (M_w_ 231,000 g/mol), an eleven-fold increase compared to when 2.3 equivalents FeCl_3_ were used. Kumar and Reynolds previously reported [26] a study of the impact of changing the amount of FeCl_3_ on the molecular weight and solubility of PEDOT-C14 when polymerized in chloroform (the order of addition of FeCl_3_ and EDOT-C14 was not specified). They found that increasing the number of equivalents of FeCl_3_ from two to four approximately doubled the degree of polymerization, from 33 to 67. These X_w_ values are considerably lower than values we obtained via standard addition in chlorobenzene (Entries 3 and 4 in Table 3). Increasing equivalents of FeCl_3_ in the standard addition synthesis of PBHOT resulted in an increase in X_w_ from <18 (M_w_ <5200 g/mol) to 99 (M_w_ 28,000 g/mol) for the 24 h experiment. This is significantly higher than the X_w_ values seen for reverse addition synthesis in chloroform of PBPOT (X_w_ = 38) and PBOOT (X_w_ = 34) reported by Qi et al. [38] Thus, this standard addition method is preferred for producing ether-substituted polythiophenes with degrees of polymerization comparable to those of P3HT. 

The smaller increase in X_w_ observed for PBHOT compared to PEDOT-C12 is likely due to steric interactions between side groups, which limit the molecular weight of polymers prepared from 3,4-disubstituted monomers, such as 3,4-BHOT [20,38,51,52,53]. PEDOT derivatives, such as PEDOT-C12, do not suffer from these issues because their fused ring structure effectively “pins back” substituent groups in such a way that steric interactions between neighboring monomers are reduced [20,30]. To determine if the X_w_ of PBHOT could be increased further, the length of the polymerization reaction was doubled from 24 h to 48 h. The longer reaction had a positive effect on X_w_, resulting in a three-fold increase to 318 (M_w_ 90,000 g/mol) over the 24 h case. Contrary to the ether-substituted polymers, an unexpected decrease in X_w_ by 93 repeat units to 395 (M_w_ 65,000 g/mol) was observed for P3HT when four equivalents FeCl_3_ were used. Extending the reaction time to 48 h increased the yield of P3HT but had no observed effect on X_w_, with both 24 and 48 h cases producing nearly identical molecular weights.

It should be mentioned that in most instances, using the standard order of addition had a negative impact on yields. This decrease in yields may be due to the acetonitrile present in the reaction mixture, even though the total acetonitrile volume is only approximately 15% (*v*/*v*) of the solution. A byproduct of the reaction, FeCl_2_, is much more soluble in acetonitrile (3 × 10^−2^ M) than in conventionally used halogenated solvents (5 × 10^−14^ M for chloroform) [36]. The much higher concentration of FeCl_2_ dissolved in the reaction solvent results in a decrease in the oxidation potential of the solution, which can suppress the oxidation of monomers and oligomers and hinder the growth of new polymer chains, thus reducing yields [36,41]. The reduced oxidation potential of the acetonitrile/chlorobenzene mixture likely results in the polymers having a larger methanol-soluble (monomer/oligomer) fraction, which is removed during purification resulting in a decrease in isolated yields. This was especially apparent for PBHOT, which suffered the largest reduction in yield. For PBHOT synthesized using standard addition, a fine precipitate that appeared in the methanol filtrate after the solutions were left undisturbed for several hours was evidence of the presence of a considerable fraction of methanol-soluble material. However, for the polymers in Entry 6 in Table 3, a different work-up procedure was used. For this case, the final product was obtained by removal of the solvent under reduced pressure instead of the usual precipitation and filtration due to solubility issues, which likely contributed to the higher isolated yield. 

To explain the different polymerization behavior observed for the ether-substituted polymers compared to alkyl-substituted P3HT, we first consider the oxidation potential of the parent monomers. The oxidation potentials of 3-alkylthiophenes, such as 3-hexylthiophene, are typically around 1.3 V vs. Ag/Ag^+^ [54], while alkyl-substituted EDOTs, such as EDOT-C12, and 3,4-dialkoxy-substituted thiophenes, such as 3,4-BHOT, are typically around 0.9 V [55] and 1.1 V vs. Ag/Ag^+^ [56], respectively. Because the oxidation potential of 3-hexylthiophene is higher than the ether-substituted monomers, it may be necessary to maintain a higher solution potential during polymerization to obtain high X_w_ for P3HT, whereas polymerization of monomers with lower oxidation potential, such as EDOT-C12 and 3,4-BHOT, may still be effective even when the oxidation potential of the solution is reduced.

Next, we consider the oxidation potential of the reaction solution, and its potential impact on the mechanism of polymerization (summarized in Table 4). When reverse order of addition is used, the oxidation potential of the solution is high because the solvent keeps the concentration of dissolved FeCl_2_ low, and the relative oxidant/monomer ratio is high. These conditions should increase the step-growth character of the polymerization mechanism because all species in solution (monomers, oligomers, and polymers) can be oxidized and participate in polymerization reactions. In a step-growth mechanism, high yields and low molecular weights are typical, which coincides with what is observed for PEDOT-C12 and PBHOT. On the other hand, when the standard addition method is used, the oxidation potential of the solution is low because the binary solvent mixture allows for a much greater concentration of dissolved FeCl_2_, and the relative oxidant/monomer ratio is kept low. These conditions should increase the chain-growth character of the polymerization mechanism, where the oxidation of longer polymer chains is favored over monomers and oligomers, which have a higher oxidation potential. Thus, polymer growth occurs by the consecutive addition of monomers to active (oxidized) polymer chains, resulting in low yields but high molecular weights, which is observed for PEDOT-C12 and PBHOT. The kinetics of the FeCl_3_-initiated polymerization of thiophene in chloroform and acetonitrile have been studied by Olinga and François [41], who reported the polymerization mechanism had some degree of solvent-dependence.

While the trends described in Table 4 describe what is observed for the ether-substituted polymers, they do not reflect what is seen for P3HT. X_w_ and yields decrease for P3HT when standard addition is used instead of reverse addition, likely due to effect of the different polymerization solvents [36]. The stark contrast in polymerization behavior between the ether-substituted polymers and P3HT suggests electronic factors (the oxidation potential of the monomer and solution) have the largest impact on the X_w_ obtained. Therefore, these factors should be considered first when seeking to optimize the polymerization conditions to different monomers. From the similar behavior observed for both PEDOT-C12 and PBHOT, it can be concluded that steric hindrance slows the polymerization reaction but does not significantly change the polymerization behavior. Reverse addition does not result in a high degree of polymerization for the electron-rich ether-substituted thiophenes [31,32]. Therefore, standard addition should be considered the method of choice for chemical oxidative polymerization of ether-substituted polymers if a high degree of polymerization is desired.

It is important to note that GPC only provides insight into the chloroform-soluble fraction of the sample. Because almost all polymerizations were carried out in chlorobenzene and all GPC experiments were conducted using chloroform, higher X_w_’s could be reached during polymerization but not dissolved during GPC sample preparation. With this in mind, we also consider the possibility that the low observed X_w_ for PEDOT-C12 and PBHOT when reverse addition is used is because a large fraction of chloroform-insoluble (very high M_w_) material is formed. This material would be filtered out from the sample prior to GPC analysis, leaving behind only the fraction of soluble, lower M_w_ material. This has been reported for the tetradecyl-substituted PEDOT-C14 (see Table 1), where the chloroform-insoluble fraction increases with the equivalents of FeCl_3_ used, with five equivalents producing material that is completely insoluble in common organic solvents [26]. To overcome the limitations of GPC and gain a clearer understanding of the effect of order of addition method on the molecular weight distribution and X_w_ of these polymers, more in-depth studies are needed. Future experiments should look to fractionate the polymers into various M_w_ ranges and quantify the relative proportion of each fraction. As well, further work needs to be conducted to optimize the reaction/purification conditions to improve yields, and computational efforts to model the dynamics of changing oxidation state as a function of solvent and reaction progression would be helpful.

## 4. Conclusions

FeCl_3_-initiated oxidative polymerization was carried out under various conditions for ether-substituted thiophenes EDOT-C12 and 3,4-BHOT along with alkyl-substituted 3-hexylthiophene. The M_w_ and X_w_ of each polymer were determined relative to polystyrene using GPC. Conditions where the oxidation potentials of the solutions were reduced (standard addition method) were found to produce ether-substituted polythiophenes with M_w_ and X_w_ higher than those synthesized using the common reverse addition method, provided they are not sterically hindered. Alternatively, conditions where the oxidation potential of the solution is kept high (common reverse addition method) were most suitable for producing high M_w_ and X_w_ P3HT. The difference in polymerization behavior is likely due to the large difference in the oxidation potential of the ether-substituted monomers compared to 3-hexylthiophene. The X_w_ data along with the isolated yields of the polymers indicate that electronic factors, such as the oxidation potential of the monomer and solution, have the greatest impact on the polymerization behavior and X_w_ obtained and thus should be carefully considered when optimizing the reaction conditions for different monomers.

## Figures and Tables

**Table 2:** Impact of different reaction parameters on the FeCl_3_-initiated oxidative polymerization of thiophenes.

Reaction Parameter	Effect	Mechanism	Ref.
Reduced Temperature	Improvement of Ð with slight decrease in yield.	Suppression of active (oxidized) monomers in favor of dimers/oligomers.	[18,40]
Improved Solvent	Increased molecular weight and improved regioregularity.	Improved solvation of polymer.	[40]
Reduced Monomer Concentration	Increased molecular weight and improved regioregularity.	Suppression of dimer/oligomer couplings and improved solvation of polymer.	[18]
Decreased Oxidant/Monomer Ratio	Yields are severely decreased, molecular weight sharply decreases (sub-stoichiometric ratio).	Overall reduction in number of oxidized species present.	[16]

**Table 3:** Weight-average molecular weight (M_w_) in g/mol and weight-average degree of polymerization (X_w_) for polymers synthesized by FeCl_3_-initiated oxidative polymerization under different conditions. M_w_ values were determined from the elution volume of the most intense peak of the GPC elugram.

Entry	Structure	Acronym	Order of Addition	Equivalents FeCl_3_	Reaction Time	Yield (%)	M_w_ (g/mol)	X_w_
1	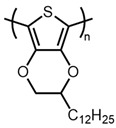	PEDOT-C12	Reverse	2.3	24 h	73	6300	20
2			Reverse ^1^	2.3	24 h	80	<5200 ^3^	<17
3			Standard	2.3	24 h	26	20,000	65
4			Standard	4	24 h	58	231,000	747
5	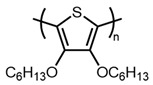	PBHOT	Reverse	2.3	24 h	42	<5200 ^3^	<18
6			Standard	2.3	24 h	78 ^2^	<5200 ^3^	<18
7			Standard	4	24 h	11	28,000	99
8			Standard	4	48 h	10	90,000	318
9	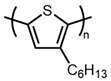	P3HT	Reverse	2.3	24 h	75	150,000	904
10			Standard	2.3	24 h	26	81,000	488
11			Standard	4	24 h	25	65,000	395
12			Standard	4	48 h	72	65,000	395

^1^ Chloroform was used as a solvent instead of chlorobenzene. ^2^ Different work-up procedure (see experimental section). ^3^ Below the lower limit of the calibrated region of the analytical column.

**Table 4:** Summary of differences between orders of addition and the trends observed for the ether-substituted polymers.

	Reverse Addition	Standard Addition
Solvent Composition	Pure chlorobenzene	Binary mixture of chlorobenzene and acetonitrile
[FeCl_2_] in Solution	Low	High
Relative Oxidant/Monomer Ratio	Begins high and decreases to theoretical value	Begins low and increases to theoretical value
Oxidation Potential of Solution	High	Low
Polymerization Mechanism	More step growth-like	More chain growth-like
X_w_	Low	High
Yield	High	Low

## Data Availability

Data is contained within the article or Supplementary Materials.

## Supplementary Materials

The following are available online at https://www.mdpi.com/article/10.3390/ma14206146/s1: detailed monomer characterization and GPC experiment information, Figure S1: ^1^H NMR spectrum of EDOT-C12, Figure S2: ^1^H NMR spectrum of 3,4-BHOT, Figure S3: APCI-MS spectrum of EDOT-C12, Figure S4: GPC calibration curve, Figure S5: GPC elugrams for PEDOT-C12, Figure S6: GPC elugrams for PBHOT, Figure S7: GPC elugrams for P3HT.
