# Supplementary material for: Enabling Conducting Polymer Applications: Methods for Achieving High Molecular Weight in Chemical Oxidative Polymerization in Alkyl- and Ether-Substituted Thiophenes
Source: Materials (Basel). 2021 Oct 16;14(20):6146. doi: 10.3390/ma14206146 (PMC8541565; doi:10.3390/ma14206146)
Supplement: Supplementary file 1 [file materials-14-06146-s001.zip › materials-1393928-SI.pdf]

materials

Article

Supplementary Information

Enabling Conducting Polymer Applications: Methods for Achieving High Molecular Weight in Chemical Oxidative Polymerization in Alkyl- and Ether-Substituted Thiophenes

David Hebert [1], Michael Naley [2], Carter Cunningham [1], David Sharp [1], Emma Murphy [1], Venus Stanton [1], and Jennifer Irvin [1,3]

[1] Department of Chemistry and Biochemistry, Texas State University, San Marcos, TX, 78666
[2] Department of Biology, Texas State University, San Marcos, TX, 78666
[3] Materials Science, Engineering and Commercialization Program, Texas State University, San Marcos, TX, 78666
* Correspondence: jennifer.irvin@txstate.edu

1. Monomer Characterization

Structural characterization of the monomers was performed using nuclear magnetic resonance (NMR) spectroscopy and mass spectrometry. ^{1}H NMR spectra were collected over 32 scans using a Bruker Avance 400 MHz NMR spectrometer with deuterochloroform as the solvent. Atmospheric-pressure chemical ionization mass spectra (APCI-MS) were collected using a Thermo-Scientific Velos Pro mass spectrometer equipped with a dual linear ion trap operating in positive mode. Monomer solutions for mass analysis were prepared in 1:1 v/v methanol/dichloromethane and diluted with additional 1:1 v/v methanol/dichloromethane to a concentration of ~20 μM by serial dilution.

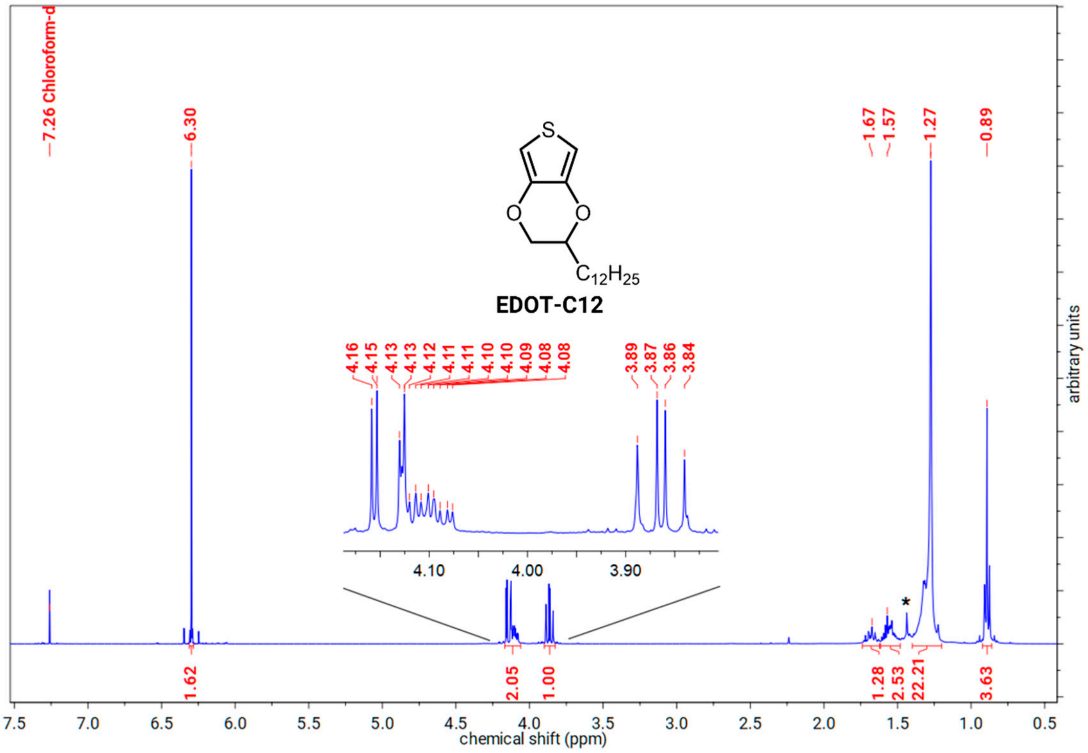

Figure S1. ^{1}H NMR spectrum of EDOT-C12. The peak labelled '*' corresponds to cyclohexane.

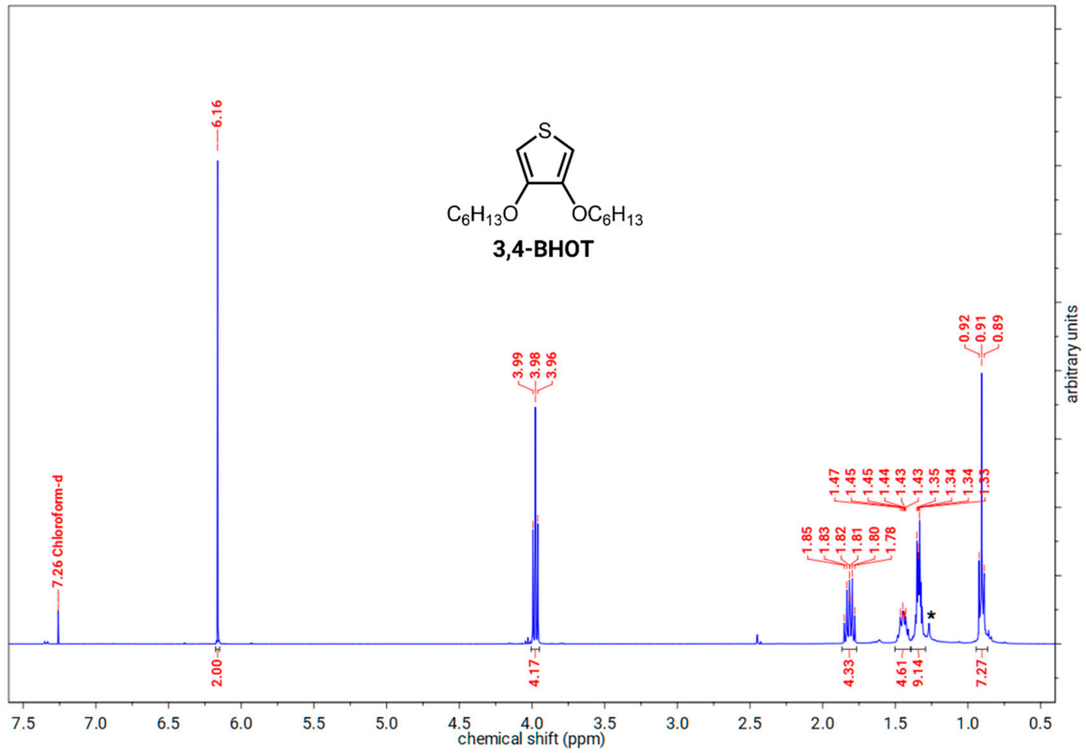

Figure S2. ^{1}H NMR spectrum of 3,4-BHOT. The peak labelled '*' corresponds to hexane.

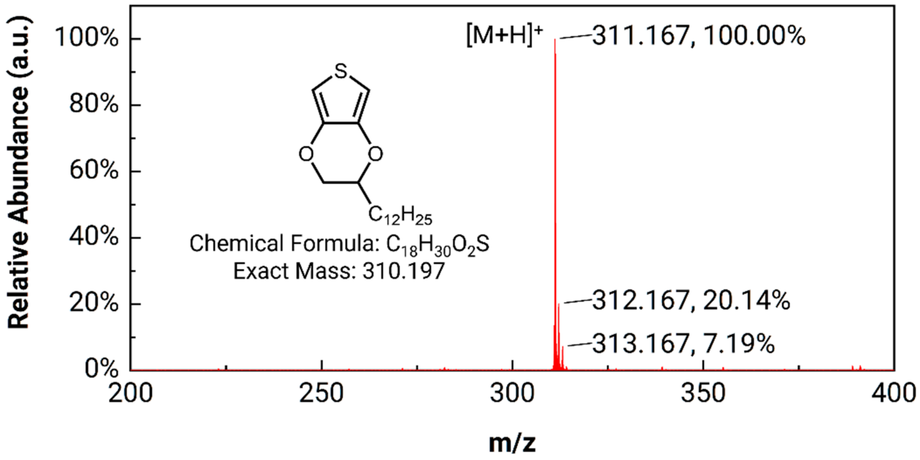

Figure S3. APCI-MS spectrum of EDOT-C12.

2. Polymer Molecular Weight Characterization

2.1 Gel Permeation Chromatography (GPC)

The molecular weight distribution of the chloroform-soluble fraction for each sample of reduced polymer was characterized by gel-permeation chromatography (GPC) using chloroform as an eluent. Samples were injected through a VE 1122 solvent delivery system and flowed at 1 mL/min through a porous styrene divinylbenzene copolymer column (Viscotek LT4000L) with RI detector (Viscotek VE3580). Molecular weights were determined relative to narrowly disperse polystyrene standards (Viscotek). Raw RI detector response data were normalized from 0-1 (baseline = 0, height of tallest peak = 1) using Origin(Pro) software (OriginLab Corporation, Northampton, MA, USA).

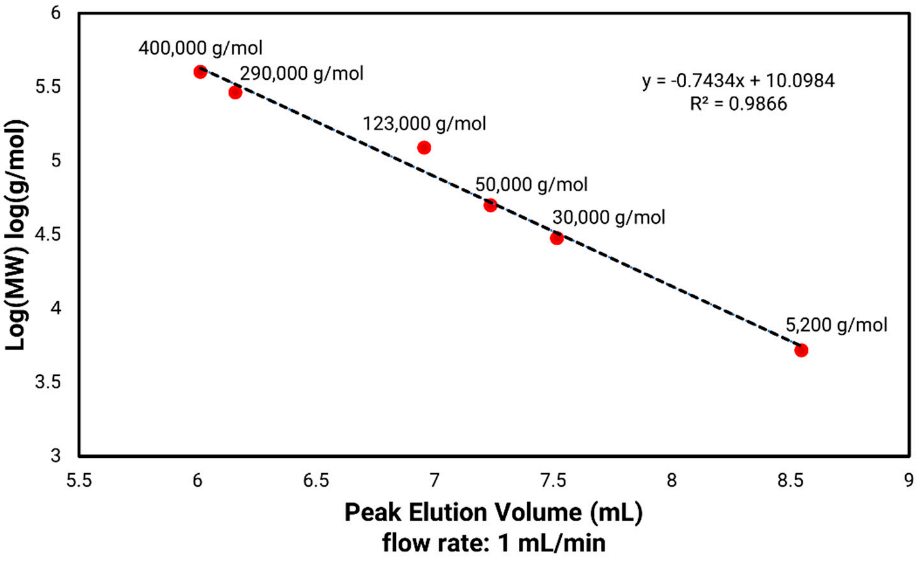

Figure S4. RI detector GPC calibration curve using polystyrene standards.

2.2 GPC Sample Preparation

Chloroform readily decomposes upon exposure to air [1] and light [2] to form phosgene and HCl. Care must be taken to eliminate these as they can dope the neutral polymers to the oxidized state, reducing their solubility. Basified chloroform was prepared by shaking repeatedly with portions of saturated aqueous $NaHCO_3$ solution until the washings remained basic to pH paper. The chloroform was dried over anhydrous $MgSO_4$ and filtered, then used immediately for GPC sample preparation.

A small sample of polymer was weighed into a 50 mL scintillation vial and diluted to a concentration between 0.1-0.25 mg/mL with freshly basified chloroform under argon. The sample was left undisturbed (stirring caused much of the sample to remain stuck to the vial walls) for 24 h at room temperature in the dark. After this period, the sample was gently shaken for a few minutes. Immediately before analysis, a small aliquot (~1 mL) of the sample was filtered through a 0.2 μm PTFE syringe filter into a clean and dry vial. The filtered aliquot was then used for analysis.

2.3 GPC Elugrams

Normalized GPC elugrams for PEDOT-C12 are shown in Figure S5. RI detector elugrams of PEDOT-C12 synthesized under different conditions. The RI signals are normalized from 0 (baseline) to 1 (height of tallest peak). Each elugram is offset vertically by 1 unit.The peak elution volume for the polymers prepared using reverse order of addition (red and gray traces) are similar, with the polymer prepared in chlorobenzene (red trace) eluting at 8.47 mL (6,300 g/mol) and the polymer prepared in chloroform (gray trace) eluting at 8.62 mL (<5,200 g/mol). However, for the polymer prepared in chlorobenzene a broad secondary peak is observed in the elugram from 5 to 8 mL (>400,000 to 14,000 g/mol), indicating there is some higher M_w material present in the sample. In contrast, the polymer prepared in chloroform shows a trailing secondary peak corresponding to low M_w oligomers. The effect of order of addition on X_w can be seen by the shift in peak elution volume to lower values from the reverse addition (8.47 mL, 6,300 g/mol, red trace) to the standard addition (7.81 mL, 20,000 g/mol, blue trace) case. Both reverse and standard addition samples encompass a similar elution volume range (*ca.* 6.5-8.5 mL, 185,000-6,000 g/mol) meaning a similar range of molecular weights are present in both samples, albeit in different relative concentrations. The polymer prepared with 4 molar equivalents $FeCl_3$ using the standard addition method (green trace) has significantly higher X_w than the other samples, which is evidenced by its greatly reduced peak elution volume (6.37 mL, 231,000 g/mol) compared to the other elugrams.

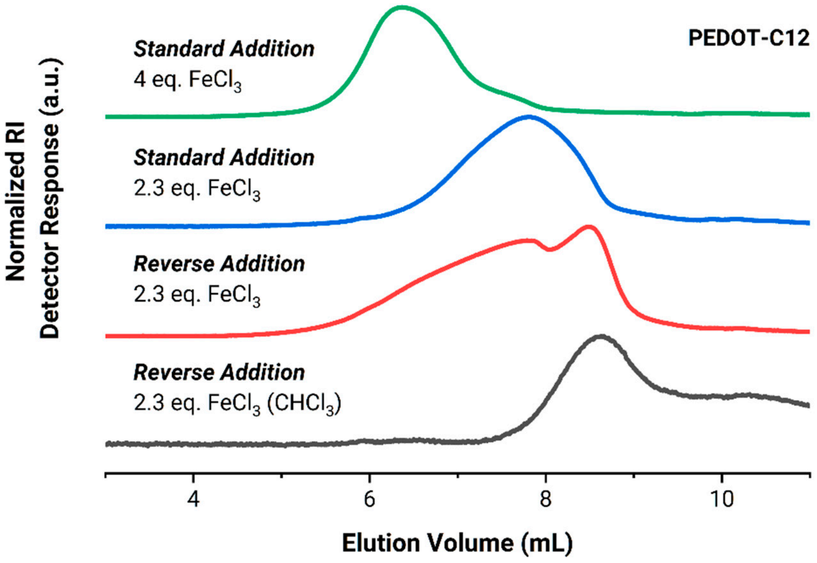

Figure S5. RI detector elugrams of PEDOT-C12 synthesized under different conditions. The RI signals are normalized from 0 (baseline) to 1 (height of tallest peak). Each elugram is offset vertically by 1 unit.

The normalized GPC elugrams for PBHOT are shown in Figure S6. The effect of order of addition on the X_w of PBHOT was less pronounced than for PEDOT-C12. The elugrams of PBHOT prepared with 2.3 equivalents $FeCl_3$ using standard (red trace) and reverse

addition (gray trace) both had a peak elution volume of 8.84 mL. The elution volume of these polymers was greater than that of the smallest M_w polystyrene standard used, indicating it had M_w <5,200 g/mol relative to polystyrene. PBHOT prepared using standard addition with 4 equivalents $FeCl_3$ (blue trace) had a complex multimodal distribution, with a peak elution volume at 7.60 mL (28,000 g/mol). The nearly flat portion of this elugram from 6.50 mL (185,000 g/mol) to the peak at 7.60 mL (28,000 g/mol) indicates there is a broad range of M_w in the sample present in similar concentration. Interestingly, extending the length of the polymerization reaction to 48 h resulted in a narrower and greatly simplified molecular weight distribution (green trace). The peak elution volume of this polymer was 6.92 mL (90,000 g/mol).

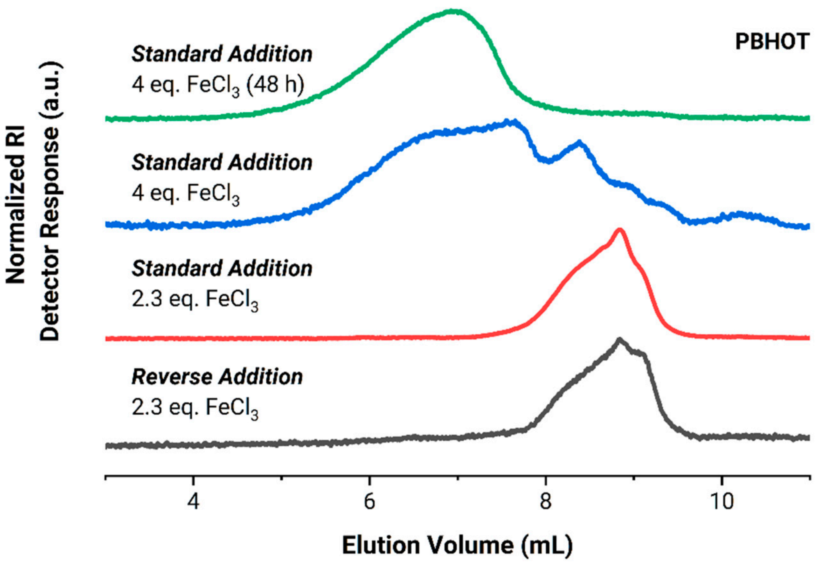

Figure S6. RI detector elugrams for PBHOT synthesized under different conditions. The RI signals are normalized from 0 (baseline) to 1 (height of tallest peak). Each elugram is offset vertically by 1 unit.

The normalized GPC elugrams for P3HT are shown in Figure S7. Increased noise in these elugrams is likely due to low analyte concentrations resulting from insoluble higher molecular weight fractions being removed via filtration during sample preparation. In contrast with ether-substituted PEDOT-C12 and PBHOT, reverse order of addition resulted in a significant increase in X_w P3HT, almost double that of the standard addition method, and changing oxidant equivalents and reaction time had minimal impact on X_w. The reverse addition polymer exhibited a bimodal molecular weight distribution (gray trace). The most intense peak had an elution volume of 6.62 mL (150,000 g/mol), and the smaller secondary peak had an elution volume of 5.95 mL, which is above the exclusion limit of the analytical column (>400,000 g/mol). When standard order of addition was used with 2.3 molar equivalents $FeCl_3$ (red trace), a slight increase in peak elution volume (6.98 mL, 81,000 g/mol) was observed, indicating a decrease in X_w. A similar decrease was observed when the standard addition method was used with 4 equivalents $FeCl_3$. The polymers prepared using these conditions for either 24 or 48 h had nearly identical peak elution volumes (7.11 mL, 65,000 g/mol) and distributions (blue and green traces, respectively).

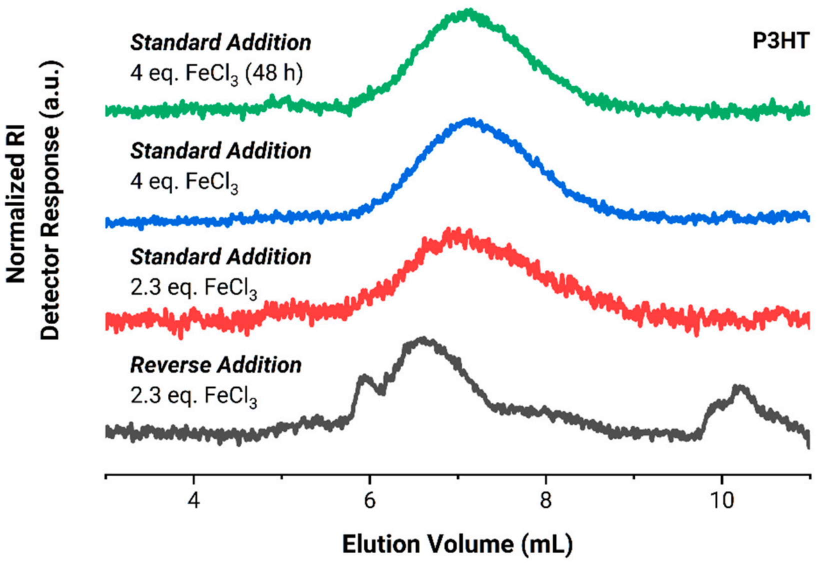

Figure S7. RI detector elugrams for P3HT synthesized under different conditions. The RI signals are normalized from 0 (baseline) to 1 (height of tallest peak). Each elugram is offset vertically by 1 unit.

References

1. Clover, A.M. The auto-oxidation of chloroform. *J. Am. Chem. Soc.* **1923**, *45*, 3133–3138, doi:10.1021/ja01665a048.
2. Hill, D.G. Photochemical decomposition of chloroform. *J. Am. Chem. Soc.* **1932**, *54*, 32–40, doi:10.1021/ja01340a004.
